# Novel Location-Based Survey Using Cognitive Interviews to Assess Geographic Networks and Hotspots of Sex and Drug Use: Implementation and Validation Study

**DOI:** 10.2196/45188

**Published:** 2023-06-22

**Authors:** Sean C Reid, Vania Wang, Ryan D Assaf, Sofia Kaloper, Alan T Murray, Steven Shoptaw, Pamina Gorbach, Susan Cassels

**Affiliations:** 1 Department of Geography University of California, Santa Barbara Santa Barbara, CA United States; 2 Benioff Homelessness and Housing Initiative, Center for Vulnerable Populations Department of Medicine University of California, San Francisco San Francisco, CA United States; 3 Family Medicine and Psychiatry and Biobehavioral Sciences University of California, Los Angeles Los Angeles, CA United States; 4 Department of Epidemiology University of California, Los Angeles Los Angeles, CA United States

**Keywords:** networks, sexual network geography, activity space, HIV, survey design, risk hotspots, cognitive interviews, health interventions, mobile phone

## Abstract

**Background:**

The Ending the HIV Epidemic initiative in the United States relies on HIV hotspots to identify where to geographically target new resources, expertise, and technology. However, interventions targeted at places with high HIV transmission and infection risk, not just places with high HIV incidence, may be more effective at reducing HIV incidence and achieving health equity.

**Objective:**

We described the implementation and validation of a web-based activity space survey on HIV risk behaviors. The survey was intended to collect geographic information that will be used to map risk behavior hotspots as well as the geography of sexual networks in Los Angeles County.

**Methods:**

The survey design team developed a series of geospatial questions that follow a 3-level structure that becomes more geographically precise as participants move through the levels. The survey was validated through 9 cognitive interviews and iteratively updated based on participant feedback until the saturation of topics and technical issues was reached.

**Results:**

In total, 4 themes were identified through the cognitive interviews: functionality of geospatial questions, representation and accessibility, privacy, and length and understanding of the survey. The ease of use for the geospatial questions was critical as many participants were not familiar with mapping software. The inclusion of well-known places, landmarks, and road networks was critical for ease of use. The addition of a Google Maps interface, which was familiar to many participants, aided in collecting accurate and precise location information. The geospatial questions increased the length of the survey and warranted the inclusion of features to simplify it and speed it up. Using nicknames to refer to previously entered geographic locations limited the number of geospatial questions that appeared in the survey and reduced the time taken to complete it. The long-standing relationship between participants and the research team improved comfort to disclose sensitive geographic information related to drug use and sex. Participants in the cognitive interviews highlighted how trust and inclusive and validating language in the survey alleviated concerns related to privacy and representation.

**Conclusions:**

This study provides promising results regarding the feasibility of using a web-based mapping survey to collect sensitive location information relevant to ending the HIV epidemic. Data collection at several geographic levels will allow for insights into spatial recall of behaviors as well as future sensitivity analysis of the spatial scale of hotspots and network characteristics. This design also promotes the privacy and comfort of participants who provide location information for sensitive topics. Key considerations for implementing this type of survey include trust from participants, community partners, or research teams to overcome concerns related to privacy and comfort. The implementation of similar surveys should consider local characteristics and knowledge when crafting the geospatial components.

## Introduction

### Background

HIV continues to disproportionately affect gay, bisexual, and other men who have sex with men (MSM) in the United States, especially young Black and Hispanic men [1-4]. Ending the HIV Epidemic in the United States is an initiative sponsored by the Centers for Disease Control and Prevention to substantially reduce HIV incidence and achieve health equity. One of the key strategies of the plan is to geographically target new resources, expertise, and technology to places with the highest HIV incidence, or HIV hotspots. A *risk hotspot* denotes areas of elevated transmission efficiency or a higher risk of disease acquisition, but most studies identifying hotspots for HIV do so through simple mapping of identified cases of disease [5]. However, interventions should be geographically targeted to places with high HIV transmission and acquisition risk, not just places with high HIV incidence. Especially for MSM, sexual, social, and residential neighborhoods are often very different places [6,7]. If an intervention is targeted at a place in which an individual with HIV lives, which might not be a place in which he engages in public life, the intervention is unlikely to be effective.

Activity spaces are a set of locations in which an individual is routinely exposed [8], such as the network, social, environmental, and political factors that may influence the risk of HIV transmission. A key contribution from place-based theory and research is that MSM are exposed to structural factors in nonresidential spaces [9-13]. These nonresidential exposures are likely critical in health research as MSM routine behaviors may be separate from HIV risk or health-seeking behaviors because of stigma or the spatial distribution of resources [14-18]. Activity space frameworks acknowledge the importance of residential and nonresidential exposures [19,20] and have increasingly been recognized as important in MSM sexual health research [18,21,22]. This work suggests that MSM usually socialize and have sex outside their residential neighborhood [7] but that prevention behaviors have similar spatial distributions as routine behavior [18]. Furthermore, nonresidential exposure can mediate the effects of residential exposure [23,24]. More work is needed to compare the contexts of various activity spaces for MSM and assess how HIV risk and substance use behaviors cluster in space.

Highly connected or centralized risk hotspots should be prioritized for interventions. If the assumption is that targeting geographical hotspots could disrupt the transmission networks of an entire community [25], then we need to identify which hotspots have the highest potential geographic and sexual network connectivity. Very little work has evaluated whether interventions based on prioritized hotspots could disrupt transmission [25,26]. Geographic research on sexual networks and HIV has identified spatial clusters of HIV cases [14,15,27-30], core groups of transmitters [31], geographic connectedness [32] or bridging across populations [33,34], and the density of networks [16,35], which can lead to more efficient transmission. Other work has examined the social context of sexual networks, often to examine disparities in HIV among heterosexual individuals [36].

Mostly missing from activity space research are geographic measures of sexual partnerships. HIV is transmitted through sexual networks that links people living with HIV to people who are susceptible to HIV, and the structure of the network influences the magnitude and timing of an epidemic [37,38]. To date, network research has explored critical features of networks, such as assortativity (partners selected based on shared attributes) and concurrency (having 2 or more overlapping partners), and how these structural characteristics affect the risk of HIV acquisition and population-level HIV transmission [39] and determine or maintain HIV racial and ethnic disparities [38,40-42]. Only recently, the spatial relationship between sexual partners and their environment has been acknowledged as a critical component of sexually transmitted infection transmission [18,35,43-47]. Substantially less research has examined egocentric spatial measures of sexual networks, which is critical to understand the places that dyads contribute to or are exposed to. Thus, sexual network geography can be thought of as the mapping of sexual network typology. The edges (relationships) between nodes (individuals and sexual partners) can be geographically referenced in space and time [17]. The places to which the individual and dyad contribute or are exposed are then the individual and dyad activity spaces [43].

### Objectives

The overarching aim of our work was to establish innovative ways to determine and prioritize geographic risk hotspots for HIV intervention among Black and Latinx MSM living in Los Angeles. In this study, we described the implementation and validation of a web-based activity space survey of HIV risk to collect the geographic information needed to visualize and identify risk hotspots and sexual network geography. The geospatial component of the survey is a custom mapping application that allows individuals to mark the exact locations of places and activities. The sensitive nature of these data warranted the validation of the geospatial survey instrument through cognitive interviews. The refinement of the survey instrument and assessment through the interviews demonstrated the validity of collecting sensitive user-defined geographic information and assessing its strengths and weaknesses. In this paper, we describe the development of the survey, the geospatial questions, the study population, and the eligibility and recruitment procedures. We then describe the cognitive interviews and how they were used to validate and update the survey instrument. Finally, we discuss the implications of our findings and how the survey is being deployed to the larger study population.

## Methods

### Overview

The study outlined in this paper is a substudy conducted by the University of California, Santa Barbara (UCSB) in partnership with researchers at the University of California, Los Angeles (UCLA). The larger parent study, mSTUDY, is an institutional review board (IRB)–approved research initiative to examine MSM at risk of HIV and substance use in the Los Angeles area. Participants agree to routine 6-month follow-ups over a 5-year period and consent to be contacted for supplemental surveys related to the goals of mSTUDY. All interactions with study participants are conducted by UCLA researchers, and the resulting data are deidentified and provided to UCSB researchers for analysis and secured data storage. The UCSB-led study outlined in this paper was intended to offer insights into the location of behavior to better inform geographically appropriate health intervention strategies through hotspot mapping and network connectivity analysis.

### Eligibility and Recruitment

The study population will ultimately consist of up to 250 Black and Hispanic MSM living in Los Angeles who are enrolled in the UCLA mSTUDY. Eligibility for this substudy was based on the same criteria for mSTUDY, which has been described in more detail in other publications [48-50]. The primary criteria of interest are being assigned male at birth, being aged between 18 and 45 years, having male sexual partners, being willing to participate in 6-month follow-up appointments, and being HIV-positive OR reporting condomless anal intercourse in the previous 6 months. A total of 2 key additional criteria relevant to this substudy were that the participants must be currently living in Los Angeles County and have access to a device with internet capabilities. Recruitment procedures were conducted by mSTUDY staff via email, SMS text message, or another suitable electronic method to comply with COVID-19 safety protocols. The recruitment protocol was the same for the cognitive interviews and full survey deployment. Substudy participants were compensated with US $40 for the cognitive interview and at least US $25 for completing the survey during data collection.

### Ethics Approval, Informed Consent, and Participation

This research protocol was approved by the UCSB IRB committee (9-23-0034). Approval was granted for cognitive interviews to validate the survey as well as for the full survey instrument. The first element of the survey is the informed consent page. This page details the purpose of the study as well as what participation in the substudy entails. The general risks of participating are outlined, as are potential benefits such as improving HIV intervention efforts in Los Angeles County. Participants are informed that taking part is voluntary and will not influence their treatment at any clinic where they currently receive medical care. Privacy and confidentiality are addressed in the informed consent page with explicit reference to a legal certificate of confidentiality. Data deidentification and storage are discussed—all personal identifiable information will be housed with UCLA, and UCSB will only work with deidentified data for analytical purposes. All participants who complete the survey are compensated with US $25. All individuals who participate in the cognitive interview are compensated with US $40 regardless of completion of the survey.

### The Survey

The survey comprises the following components: consent page, geospatial question tutorial, demographic questions, life event questions, mobility and housing questions, and geospatial questions. Each component is described in detail in the following sections.

#### Consent Page

The consent page outlines key information about the study aims, privacy, risks, and benefits associated with survey participation. The consent page emphasizes that participation in the substudy is voluntary and can be discontinued at any time without affecting their clinical care.

#### Geospatial Question Tutorial

The geospatial questions are the primary focus and most important instrument of the survey. These questions allow for the novel identification and understanding of sex and drug use hotspots in Los Angeles County. A tutorial is included at the beginning of the survey to ensure early exposure and comfort with these questions. The tutorial focuses on a trivial example of selecting their favorite ice cream shop to practice using the mapping features.

#### Demographic Questions

This section gathers information related to race and ethnicity, age, gender, education, employment, and family characteristics. This information is intended to supplement data from the parent mSTUDY survey.

#### Life Event Questions

The primary focus of this section is sexual orientation and identity and how it has changed over the course of their lives. The questions in this section cover topics related to sexual behavior, the number of sexual partners, and the age of first sexual experience. In addition to lifetime sexual experiences, this section includes questions on the positive and negative factors of their residential environment.

#### Mobility and Housing Questions

This section gathers information on housing instability, household composition, and modes of transportation. These questions provided insights for the analysis of the geospatial information collected later in the survey.

#### Geospatial Questions

There are several categories in this section that are tied to specific locations. The geospatial questions follow the same format described in the *Geospatial Question Tutorial* section and ask about locations relevant to mapping hotspots of sex and substance use. The topics covered in this section include current and previous home locations, school and work locations (if applicable), socialization locations, health care and testing locations, drug use locations, and sexual act locations. The questions in this section are sensitive and underscore the importance of the tutorial to ensure that participants know about their privacy options and geospatial functionality when answering these questions.

### Survey Development

Common methods for collecting geographic data include surveys that elicit self-reported addresses [33,51], web-based surveys that use web-based maps to allow participants to find and verify locations [18,22], and geographical momentary assessment methods to collect spatiotemporally referenced data via GPS-enabled smartphones or other wearable devices [52-55]. Each method has costs and benefits, balancing respondent burden, accuracy, validity, and ethical considerations. For example, web-based map surveys result in fewer errors compared with self-reported addresses as participants interact directly with the maps, but the accuracy of the data depends on participant recall and willingness to report locations of illegal or stigmatized behavior. Geographical momentary assessment methods may be more valid but are costlier and burdensome and have substantially more privacy and ethical concerns. According to a study of individuals who use substances [54], providing location information in interviewer-administered surveys would not be a concern, but the sensitivity of the question could alter accuracy.

We adapted a web-based activity space questionnaire [18] using an internet map–based questionnaire on regular activity locations [56] as it is a cost-effective, low-burden, and feasible approach for high-risk and drug-using populations and has been shown to be a more valid way to assess activity space compared with GPS-tracking methods [57]. We used questions from a web-based activity space survey originally developed by Vaughan et al [18] at Emory University. Our goal was to supplement this survey with additional geographically embedded questions regarding substance use locations and a geographically explicit sexual network module to capture information about dyad activity spaces. For each partnership, we requested information about where the respondent (ego) lived, where the partner (alter) lived, where they met (ie, on the web or an in-person location), where they last had sex, the type of last sex (condom use and position), substance use, partnership characteristics (the type of partnership and whether it is ongoing), and sexual partner characteristics (sociodemographics, HIV status, and status disclosure). In addition to partnership and substance use locations, we used the mapping features to request information on home, work and school, social, health care, and testing locations. The survey was built in Qualtrics (Qualtrics International Inc) based on its distribution features and JavaScript-based customization that facilitated mapping-based questions using Leaflet and Google Maps.

The implementation of the geospatial questions faced several key hurdles and considerations as the application was developed. The goal was to gather precise and robust geographic information while also preserving the privacy and comfort of each participant. After careful consideration, the research team chose a 3-level design where participants work their way through several maps that start with large, generalized regions and become more precise and fine-scale until the participants can select an exact location. Participants are then prompted to enter their confidence in the marked location. The move from general to specific allows participants to provide location information up to their comfort level while also providing several levels for subsequent analysis. The 3-level design allows participants to provide more generalized information in cases where they cannot remember or do not feel comfortable providing exact locations. The 3-level design addressed privacy concerns but increased the length of the survey. To account for this, the design team added a nickname component for each location. Participants are asked to give a location a nickname that is easy to remember and meaningful to them. The nicknames appear later in the survey as answer choices to reuse locations without using the geospatial component, which reduces the amount of time needed to complete the survey. The use of a geographic information system analytical framework allows for easy linkage of nicknames to locations in the data processing phase. The introduction of the geospatial questions in the survey is discussed in more detail in the following sections. Participants are shown a low-stakes example of the geospatial question so that they are familiar with the process when they encounter more sensitive questions later in the survey.

**Figure 1:**
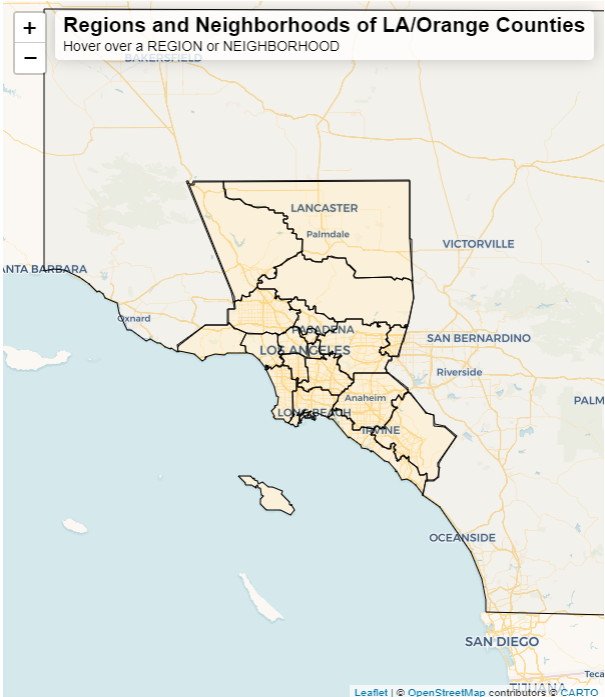
The map shows the first level of the geospatial question block. The Leaflet application was used to build and visualize the map shown in this figure. Participants are shown this map of generalized regions, shown as yellow polygons, in Los Angeles (LA) County to choose an area where a place is or an activity took place. These regions were chosen by the design team based on local knowledge of familiar regions known to residents of LA County. Participants can pan around and zoom into the map to orient themselves using the underlying base map provided by OpenStreetMap. The display bar at the top of the map will populate with the name of the region when clicked or when the mouse hovers over the region.

In the first level, participants are presented with a map (Figure 1) that shows large, commonly known regions in Los Angeles County. Examples of these regions include Central Los Angeles, Antelope Valley, San Fernando Valley, and North County. Through discussion with the study team with local knowledge of Los Angeles, we agreed that the regions included in the first level of the mapping questions should be familiar to residents. An underlying road base map was also included to assist participants in situating themselves on the map. The names of regions are shown in a display bar at the top of the map when a region is clicked or when the pointer is hovered over the region. Once participants select a region, they move to the next level of the geospatial question.

**Figure 2:**
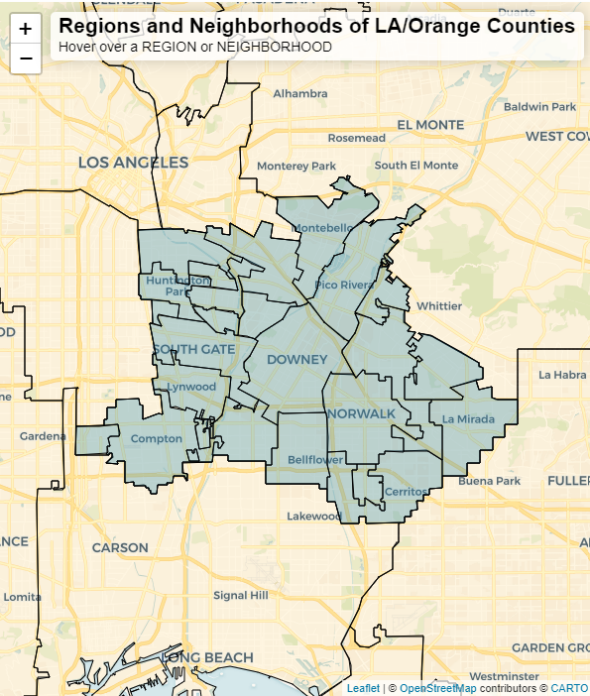
The map shows the second level of the geospatial question block. The Leaflet application was used to build and visualize the map shown in this figure. Participants are zoomed into the region they selected in the first level and are shown smaller neighborhoods, shown as blue polygons. These neighborhoods were chosen by the design team based on local knowledge of familiar neighborhoods known to residents of Los Angeles (LA) County. Participants can pan around and zoom into the map to orient themselves using the underlying OpenStreetMap base map. The display bar at the top of the map will populate with the name of the neighborhood when it is clicked or when the mouse hovers over the neighborhood.

The second-level map is a continuation of the first; the participant is zoomed into the region chosen in the first level. They are presented with smaller neighborhoods within the region (Figure 2), which were chosen based on familiarity to Los Angeles residents. Examples of these neighborhoods include West Hollywood, Downtown, Koreatown, and Silver Lake. The functionality of this map mirrors the first level but is presented at a finer geographic scale. The neighborhoods in this level are quite specific for geographic analysis while maintaining privacy for the participants. Once a neighborhood is chosen, the participant moves to the third level of the geospatial question.

**Figure 3:**
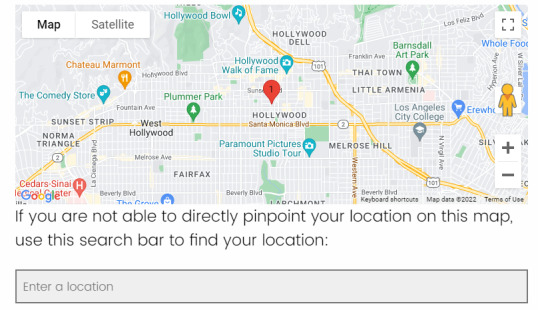
The map shows the third level of the geospatial question block. This map is generated using an embedded Google Maps interface that was chosen based on widespread use. Participants can pan around and zoom into the map as in the previous levels. This level uses a pin drop to mark an exact location on the map. Participants can move the pin by dragging it to the desired location, clicking on the desired location to automatically move the pin, or using the search bar to type in an address or name of a location to move the pin.

The third level has a different look and feel than the previous levels (Figure 3) and allows participants to mark an exact location. Participants are presented with an embedded Google Maps interface that is zoomed into the center of the neighborhood they selected in the second level. The Google Maps interface was chosen because of the wide use of the application, and the design team wanted something that felt familiar to those taking the survey. Participants can mark a location on the embedded map in three ways as follows: (1) panning around the map to orient themselves or find familiar locations before clicking on a location to move the pin on the map to that location, (2) dragging and dropping the pin on the desired location, and (3) typing in the name of a location or an address if they know it to move the pin to that location.

After the participant has marked a location, they answer an additional question to assess how they were able to interact with the mapping component in the geospatial question block. Participants provide feedback on the accuracy and precision with which they marked the question. Accuracy in this context means how sure the participant is about the location they marked. For example, they can indicate that they cannot remember the exact location but marked the general area or that, because of technical difficulties using the map, they were unable to mark an accurate location. Precision in this context means how close the participant moved the pin to the exact location. For example, they can indicate that they know the exact location but marked a place within a block of the location because of privacy concerns. The follow-up question is invaluable for subsequent geospatial analyses to allow for corrections based on uncertainty in the data.

### Cognitive Interviews

We conducted 9 cognitive interviews to validate the survey instrument and systematically review and improve the survey before sending it out to all eligible participants. The primary focus of the cognitive interviews was to improve the clarity of the consent language and the survey questions and ensure the ease of use and understanding of the geospatial questions. We evaluated each of these components using the think-aloud method during the cognitive interviews.

We recruited participants for the cognitive interviews based on the requirements outlined in the previous sections. On the basis of the IRB approval, we are unable to provide additional descriptive characteristics of the participants. The target number of interviews was 10 or until the saturation of topics related to the improvement of the survey was reached. The consent process was the same for participants in the cognitive interviews except that they must agree to participate in the interview over a videoconferencing platform—in this case, Zoom (Zoom Video Communications)—with the screen-sharing feature. This allowed researchers to observe and ask questions about the survey as well as about how participants felt interacting with it. The participants had the option to take the survey on a computer or a mobile device.

Each interview was conducted in the same manner following the protocols approved by the UCSB Human Subjects Review panel. A researcher from the UCLA mSTUDY program conducted the interview using probing questions and took the lead on all interactions with the participants in the cognitive interview. The participants consented and were informed of the purpose of the cognitive interview at the start of each interview and informed that they could stop the interview at any time. The UCLA researcher provided the participants with a link to the survey and asked them to share their screens. Each participant was asked to read the instructions for each question and try to complete the question on their own before asking for assistance. They were also asked to voice any concerns or questions that they had as they progressed through the survey. Periodically during the interview, the interviewer asked for feedback on clarity and comfort that the participants felt while taking the survey. While the interview was taking place, a researcher from UCSB observed in real time to take notes on survey performance and noted areas for improvement. After the participants completed the survey, they were debriefed and asked about their perceptions of the survey. The insights gained from the cognitive interviews resulted in alterations to the survey to improve the survey interface and participant understanding of the questions. These alterations were particularly important for the geospatial questions to ensure that participants used the mapping interface appropriately and effectively. The alterations are discussed in more detail in the following section.

## Results

### Overview

A total of 4 themes were identified from the cognitive interviews: functionality of geospatial questions, representation and accessibility, privacy, and length and understanding of the survey. Although each of these themes is important on its own, there is overlap because of the interconnected nature of the survey. The participants involved in the cognitive interviews spanned the eligibility spectrum for this study, which influenced their answers regarding each of the 4 identified themes.

### Functionality of Geospatial Questions

The geospatial component of the survey was the most novel and critical portion to be evaluated during the cognitive interviews. The tiered design of the geospatial questions described in the *Methods* section was a key area for evaluation, necessitating adjustments throughout the cognitive interviews to ensure optimal results.

From the first 4 cognitive interviews, it became apparent that participants had difficulty orienting themselves on the map in the first 2 levels of the geospatial questions. The first iteration of the survey did not have transparent regions and neighborhoods that exposed the underlying road network that many residents of Los Angeles County are familiar with. Participants were unsure of what the map was showing, and even with instructions on the page, they were not sure how to answer the question. They relied heavily on the interviewer to help them with this portion of the survey. Even with aid from the interviewer, there was frustration with this part of the survey, which resulted in some participants randomly selecting regions and neighborhoods to get through the question quickly. Common feedback on this version of the Leaflet map included being confused about the content of the map and missing landmarks needed for map orientation. Participants unanimously said that they mainly traveled around the city by car and suggested that a map with roads would improve the utility of the survey. These suggestions resulted in the implementation of transparent regions and neighborhoods that showed underlying road networks (shown in Figures 1 and 2). This change in the map greatly improved understanding for all future participants on the first 2 levels of the geospatial question as this issue did not arise in later cognitive interviews.

Participants favored using the third level of the geospatial question based on familiarity using the Google Maps interface. Participants had a much easier time navigating the map features compared with the first 2 levels of the Leaflet map. Many participants (4/9, 44%) suggested the removal of the first 2 levels altogether based on ease of use, but they were ultimately kept for privacy reasons and data robustness provided by the additional geographic levels. However, there were still errors made on the third level even with greater comfort in using the interface. Many errors involved moving the pin to an incorrect location or accidentally moving the pin when it was not intended. Pin placement also varied based on the zoom level used when navigating the map. Some participants (3/9, 33%) did not zoom into the map closely, which made moving the pin to a precise location more difficult. These erroneous pin placements caused incorrect answers to the subsequent follow-up question. Many participants (4/9, 44%) said that they had marked an exact location but the pin was not at the intended location. In addition to issues regarding pin placement, there was some confusion on how to use the search bar under the map in the third level. Some used the search bar as intended and moved through the mapping questions quickly, whereas others did not understand the utility of the search bar. The misuse of the search bar and pin did not hinder participants from completing the survey as intended, but it prompted the survey development team to include a detailed tutorial on how to use the mapping components of geospatial questions to alleviate confusion. This tutorial was added after the sixth interview, and the remaining interviews had far fewer errors when completing the geospatial questions in the survey. The errors observed using the Google Maps interface further substantiated the need for the first 2 levels of the geospatial question to provide alternative data that are more accurate but less precise.

The reception and use of nicknames at the start of each geospatial question varied widely among participants in the cognitive interviews. For some participants (3/9, 33%), the importance of using nicknames was not clear until the end of the survey, when they were used to bypass the geospatial question structure. As a result, some nicknames were vague and difficult to remember, which limited their usefulness when they appeared as answer choices later in the survey. Despite this, participants saved time moving through the survey by using the nicknames.

The technology used by each participant influenced their ability to interact with the survey. Those who used a computer were more easily able to navigate the geospatial questions, whereas those who used a mobile device experienced more technical difficulties. The size of the screen on mobile devices created difficulties in the geospatial questions. In some cases, the map was larger than the screen, and participants could not scroll past the map to move on to the next question. The first 2 levels of the geospatial questions were particularly difficult as each region had to be clicked to show the name versus hovering over the location with the mouse on a computer. This resulted in more time needed to complete the surveys and more instances of frustration when using a mobile device. The operating system and device age also posed challenges. Some devices had very old software that was not compatible with survey functionality and limited how participants could interact with the survey. In 1 case, the cognitive interview had to end because of an incompatible mobile device and the lack of access to a computer. Solutions to these issues are discussed in more detail in the *Discussion* section.

### Representation and Accessibility

We aimed to make our survey inclusive and representative for the participants. Our study population was primarily Black and Latino MSM, and the survey was crafted to reflect that. Many participants (4/9, 44%) acknowledged our survey design as being robust and representative and reiterated that it was not something they often saw in other surveys. These comments in particular came in the demographic and life event sections, where questions related to race, ethnicity, and sexual orientation were located. These comments showed a level of trust and openness that were important for sensitive questions later in the survey.

Although the representativeness of the answer options was a positive trait, some participants (2/9, 22%) initially felt overwhelmed or confused about certain terms used in the survey. This limited the understanding of the questions and the ability to answer each one confidently. Some participants (3/9, 33%) commented that they were unsure about which answer choice best represented them and suggested the removal of some terms. For the sake of inclusivity, the robust answer options in the survey were preserved in the final iteration.

### Privacy

Privacy and comfort were important considerations in the development phase of the survey, particularly regarding the design of the geospatial questions. When prompted with probing questions in the cognitive interview, some participants (3/9, 33%) shared similar concerns and asked about the reasoning for questions on sensitive behaviors and locations. Most concerns were rooted in how the data would be deidentified and stored, which could be found in the informed consent portion at the start of the survey. This indicated that many participants missed or did not understand this information in the informed consent portion; in response, we added bold and underlined headings to the consent page.

Participants were willing to enter sensitive information but had follow-up comments and questions during the cognitive interviews. Some participants (3/9, 33%) stated feeling awkward sharing personal answers such as lifetime number of sexual partners or the age of first sexual experience during the cognitive interview. However, they reiterated that they would feel more comfortable and would answer honestly when taking the actual survey on their own in private. Privacy concerns related to the geospatial questions were similar. Some participants (2/9, 22%) felt that the questions seemed like *big brother* was watching them, or they felt like a *narc* for sharing locations related to drug use and purchase. All participants (9/9, 100%) entered the requested information but felt wary at times of the novel geospatial questions. Ultimately, respondents shared the sensitive information as they understood the purpose of the survey and trusted the parent mSTUDY run by UCLA.

Other participants (5/9, 55%) did not share the privacy concerns and felt comfortable sharing sensitive location information. Some participants (3/9, 33%) felt so comfortable during the interview that they shared additional details about locations and sexual experiences to provide ideas for additional questions in the survey. They also stated that the follow-up questions on how they marked a location provided flexibility and privacy in the survey. This group of participants often cited their comfort and trust in working with mSTUDY over multiple years. Several participants (2/9, 22%) also stated that they were motivated to share additional details as they felt that their answers could improve care for MSM in Los Angeles County.

### Length and Understanding of the Survey

There was considerable variation in the level of understanding and time needed to complete the survey. The main drivers of this variation were technological difficulties, English competency, participant age, and understanding of the diverse and representative answer choices in the survey.

For some participants (4/9, 44%), technological difficulties were the greatest challenge and even hindered their ability to complete the survey. Troubleshooting the issues often required a great deal of support from the interviewer in the cognitive interview, and many of the problems may not have been resolved without aid. Higher levels of technical difficulty were noted among older participants. Feedback on technological difficulties was related to participants not using their devices often or only using them for a few specific purposes.

In interviews where the participants did not speak English as their first language, there was an increase in the time taken to complete the survey. The survey had descriptive instructions, and this was cited as a reason for the extra time needed to complete the survey. Although the diversity of answer choices was seen as a strength of the survey, it also created some confusion regarding the meaning of the words, especially among participants who did not speak English as their first language. The goal regarding survey completion time was between 30 minutes and 1 hour. The average time to complete the survey during the cognitive interviews was 53 (SD 26) minutes even with additional probing questions.

## Discussion

### Principal Findings

The primary goal of this study was to provide an innovative way to collect accurate and precise geographic information using a novel location-based survey instrument that can be used to prioritize interventions in HIV risk hotspots. Cognitive interviews were used to validate the survey instrument, and the results indicated that this data collection method is viable. After several iterations of the survey, the development team felt that the survey instrument functioned appropriately to effectively collect accurate and precise location-based information on HIV risk behavior and other routine behavior data vital for activity space–based research. Although the results from the cognitive interviews are promising, there are several key considerations that should be discussed. First, the design of the 3-level geospatial questions should be catered to the area under investigation to include common regions and landmarks that are easily recognizable to participants taking the survey. The region and neighborhood design used in this study may not be generalizable to different cities or rural areas.

The issue of trust came up throughout the cognitive interviews. Probing questions during the interviews highlighted varying levels of comfort answering the geospatial questions related to sensitive topics such as sex and drug use. All participants ultimately answered the questions and cited trust in the organization as the primary reason why they felt comfortable. This highlights the key role of trust and respect between the study population and research and community-based organizations in data collection efforts for research projects, especially regarding sensitive information. It also validates the focus the design team put into ensuring privacy, flexibility, and representative language in the survey questions themselves. The representative answer choices throughout the survey served to reinforce trust with the participants as they felt represented and understood in the survey. Without community partners and the privacy of participants being at the forefront of design decisions, the survey instrument may not have been as effective. Feedback received from participants also highlighted the need for clear and easy-to-navigate consent forms that provide relevant information needed to alleviate privacy concerns.

The complexity and functionality of the geospatial questions were important in garnering quality data from participants taking the survey. The features shown in the map were customized to show familiar places and landmarks that are needed for locals to effectively orient themselves on the map. Some participants (3/9, 33%) stated that they did not feel comfortable using unfamiliar geographic mapping features, which highlighted the importance of familiar and easy-to-use mapping features such as the Google Maps interface. The implementation of the underlying road network was an important breakthrough during the cognitive interview process and greatly improved the usability of the geospatial questions. There were several technical issues related to the map, such as zoom level and pin placement, that could not be addressed and potentially influenced data quality. This highlights the importance of the 3-level design used in the geospatial questions to ensure that usable data are collected at varying geographic resolutions. It also allows for sensitivity analysis in the identification of risk hotspots to improve the understanding of survey development using geographic data.

The level of comfort that each participant felt interacting with the geospatial questions directly influenced the amount of time taken to complete the survey. This problem was addressed using nicknames for locations that could be used in the survey to bypass the geospatial questions by recycling previously entered locations. This feature relies heavily on the participants entering meaningful nicknames that they will remember later in the survey. To alleviate the burden on participants in coming up with many meaningful nicknames, the design team auto-assigned nicknames for difficult-to-label locations such as health care, substance use, and sex act locations. The nicknames functioned well to save time in completing the survey, but it is important to note that too many nicknames can be detrimental to survey performance.

There are several limitations to consider from the cognitive interviews that must be accounted for before full deployment of the survey. The 2 most important limitations are technical difficulties related to the device participants used to take the survey and language barriers. Both resulted in frustration with the survey and the time needed to complete it, which can limit the ability and willingness of participants to complete the survey. To account for this, during the deployment of the survey, the research team devised a protocol of recommendations for participants. Troubleshooting tips and technical recommendations such as using a computer instead of a mobile device are included in recruitment emails to prepare participants for potential problems. If problems persist, participants are directed to use public resources such as a library to complete the survey or use a computer at the clinic at their next health care appointment. This again highlights the importance of working with trusted community partners who can aid in the implementation of the survey.

### Conclusions

This study shows the promise of using a novel geospatial survey instrument to collect precise geographic information needed for effective HIV intervention efforts. The effective implementation of this type of survey relies on trust from participants and community partnerships to overcome limitations. Iterative updates throughout the cognitive interviews were key for improving the survey before full deployment. Future work can examine the effectiveness of novel location-based questions related to sensitive topics from a crowdsourced population to compare with studies conducted with a community partner. This study displays initial results indicating the feasibility of collecting this type of information with a marginalized and underrepresented group in an effort to improve access to care and contribute to ending the HIV epidemic.

## Abbreviations

IRB: institutional review board
MSM: men who have sex with men
UCLA: University of California, Los Angeles
UCSB: University of California, Santa Barbara

## Data Availability

All data related to the participants, including information collected during the cognitive interviews, are strictly confidential. Access to these data is restricted to the University of California, Los Angeles and mSTUDY team to ensure the privacy and confidentiality of the participants. A copy of the survey can be made available upon request to the lead author. Additional information about participants in the cognitive interviews, such as demographic data, is not available based on the approved institutional review board guidelines.
